# *Clinacanthus nutans* (Burm. f.) Lindau Ethanol Extract Inhibits Hepatoma in Mice through Upregulation of the Immune Response

**DOI:** 10.3390/molecules200917405

**Published:** 2015-09-18

**Authors:** Danmin Huang, Wenjie Guo, Jing Gao, Jun Chen, Joshua Opeyemi Olatunji

**Affiliations:** 1 School of Food and Biological Engineering, Jiangsu University, Zhenjiang 212013, China; 2 Bio Nice Food Science Sdn. Bhd. No.5, Jalan SILC ¼, Perindustrian SILC, Nusajaya, Johor 79200, Malaysia; 3 School of Pharmacy, Jiangsu University, Zhenjiang 212013, China; E-Mails: guowj06@126.com (W.G.); jinggao@mail.ujs.edu.cn (J.G.); pere@fastermail.com (J.O.O.)

**Keywords:** *Clinacanthus nutans*, tumor, apoptosis, immunoregulation

## Abstract

*Clinacanthans nutans* (Burm. f.) Lindau is a popular medicinal vegetable in Southern Asia, and its extracts have displayed significant anti-proliferative effects on cancer cells *in vitro.* However, the underlying mechanism for this effect has yet to be established. This study investigated the antitumor and immunomodulatory activity of *C. nutans* (Burm. f.) Lindau 30% ethanol extract (CN30) *in vivo.* CN30 was prepared and its main components were identified using high-performance liquid chromatography (HPLC) and mass spectrometry (LC/MS/MS). CN30 had a significant inhibitory effect on tumor volume and weight. Hematoxylin and eosin (H & E) staining and TUNEL assay revealed that hepatoma cells underwent significant apoptosis with CN30 treatment, while expression levels of proliferation markers PCNA and *p*-AKT were significantly decreased when treated with low or high doses of CN30 treatment. Western blot analysis of PAPR, caspase-3, BAX, and Bcl2 also showed that CN30 induced apoptosis in hepatoma cells. Furthermore, intracellular staining analysis showed that CN30 treatment increased the number of IFN-γ^+^ T cells and decreased the number of IL-4^+^ T cells. Serum IFN-γ and interleukin-2 levels also significantly improved. Our findings indicated that CN30 demonstrated antitumor properties by up-regulating the immune response, and warrants further evaluation as a potential therapeutic agent for the treatment and prevention of cancers.

## 1. Introduction

Hepatocellular carcinoma (HCC) is one of the most prevalent and lethal liver malignancies, with over half a million new cases annually. Hepatitis B and C virus infections account for the majority of reported HCC cases, although other factors such as obesity, diabetes, and cirrhosis, are increasingly becoming relevant in HCC, largely due to its limited treatments [1]. Currently, only 5% of patients with HCC are suitable for transplantation or surgical resection due to the advanced stage at diagnosis in most patients [2]. Vincristine, fluorouracil, cytoxan, cisplatin, and doxorubicin are the first-line medicines for HCC treatment. However, most of these drugs are non-specifically cytotoxic and produce significant side effects [3]; because of this, there has been a search for more effective and relatively safer treatments for HCC based on natural products.

Tumor and cancerous cells express low major histocompatibility complex (MHC) levels and high levels of immune suppressive cytokines (IL-10 and TGF-β), leading to compromised immune regulatory cells. These immune suppressive cytokines have been implicated in tumor progression in many types of cancers, and cause tumor cells to be invisible to the innate immune system [4,5]. In addition to the conventional mechanisms by which anticancer agents are widely believed to act, increasing evidence suggests that anticancer agents could also exert their effects by supplanting immunosuppressive mechanisms induced in tumor cells by directly or indirectly stimulating immune effectors [5].

*Clinacanthus nutans* (*C. nutans*), also known as Sabah snake grass in Malaysia, belongs to the family of Acanthaceae and is a native herb in tropical Asia. It is an important traditional medicine in China, Malaysia, and Thailand [6]. In Malaysia and Thailand particularly, it has been widely used in the treatment of skin rashes, insect and snake bites, herpes simplex virus (HSV) and varicella-zoster virus lesions, mental tension, diabetes, and rheumatoid arthritis [7,8,9]. Previous reports have indicated that chloroform extracts from *C. nutans* (Burm. f.) Lindau displayed significant ant proliferative effects on various cancer cells *in vitro* [10]. However, the mechanism underlying this anticancer activity is yet to be understood or elucidated. Therefore, the present study aimed to explore the inhibitory effect of an ethanol extract of *C. nutans* on hepatoma *in vivo* and further investigate its underlying multiple immune-based mechanism of action.

## 2. Results and Discussion

### 2.1. Identification CN30 by HPLC

**Figure 1:**
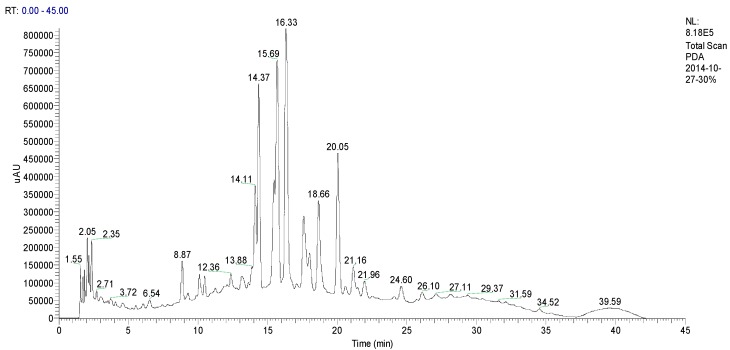
High-performance liquid chromatography (HPLC) chromatograms of the flavonoids in CN30 extract fractions purified through a C18 cartridge.

**Figure 2:**
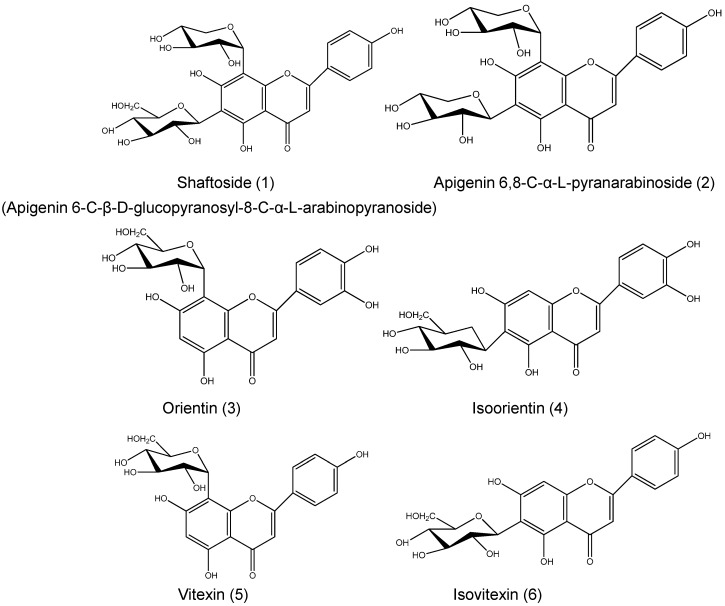
Chemical structures of known flavones in CN30 extract: (1) Shaftoside (Apigenin 6-C-β-d-glucopyranosyl-8-C-α-l-arabinopyranoside); (2) Apigenin 6,8-C-α-l-pyranarabinoside; (3) Orientin; (4) Isoorientin; (5) Vitexin; (6) Isovitexin.

**Figure 3:**
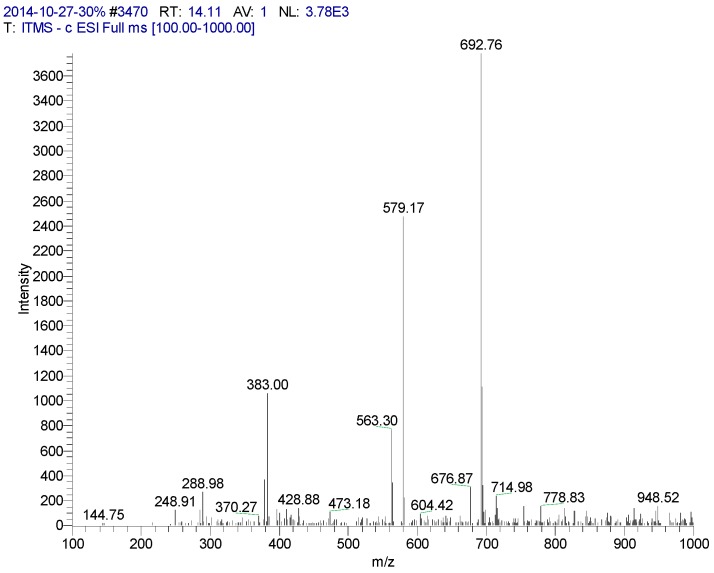
Mass spectrometry of CN30 extract by HPLC in the 14.11 min peak area.

**Figure 4:**
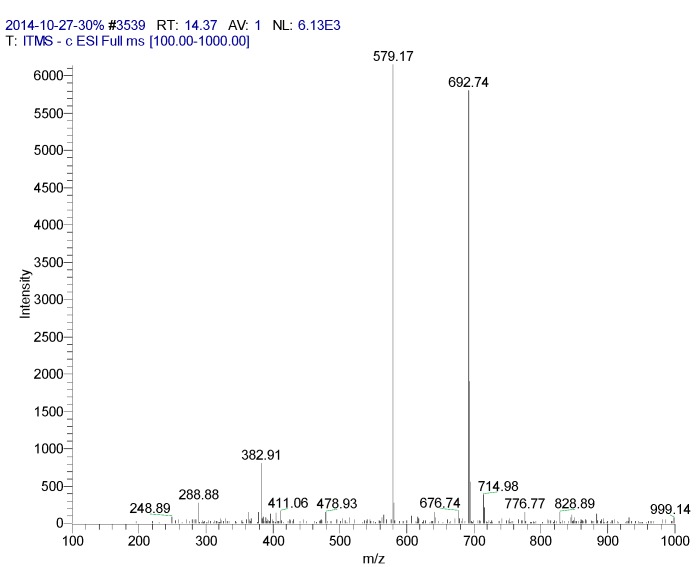
Mass spectrometry of CN30 extract by HPLC in the 14.37 min peak.

**Figure 5:**
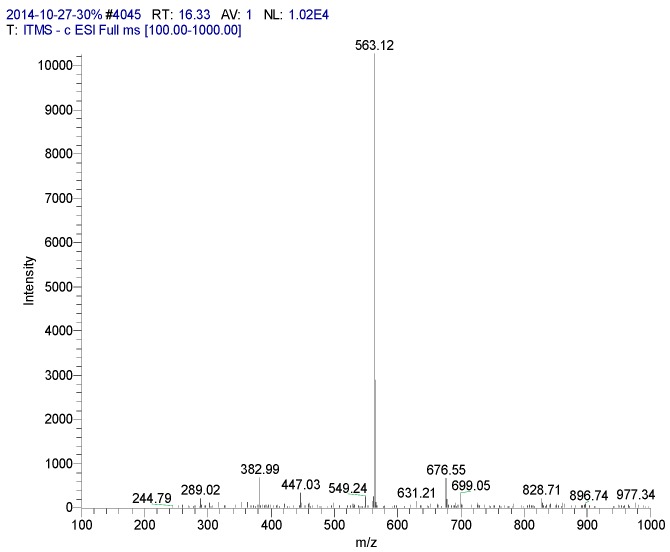
Mass spectrometry of CN30 extract by HPLC in the 16.33 min peak.

**Figure 6:**
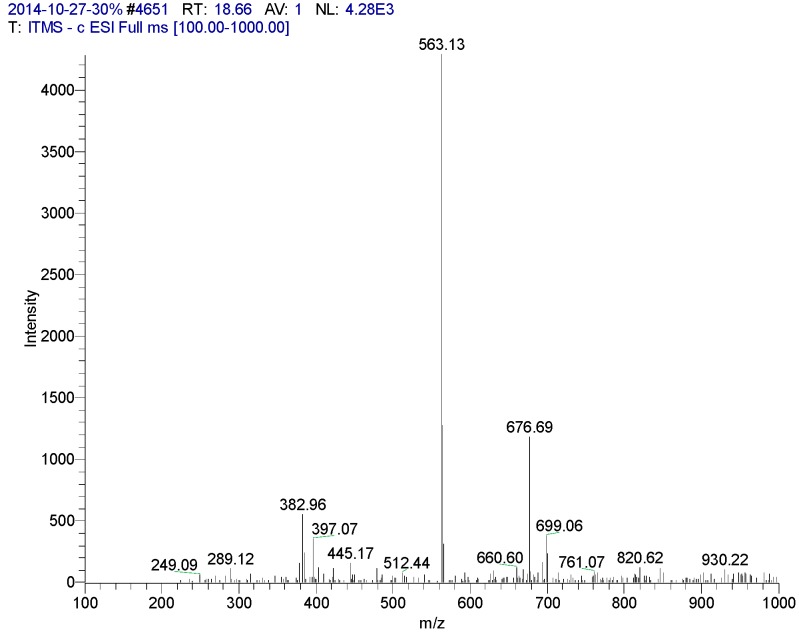
Mass spectrometry of CN30 extract by HPLC in the 18.66 min peak.

**Figure 7:**
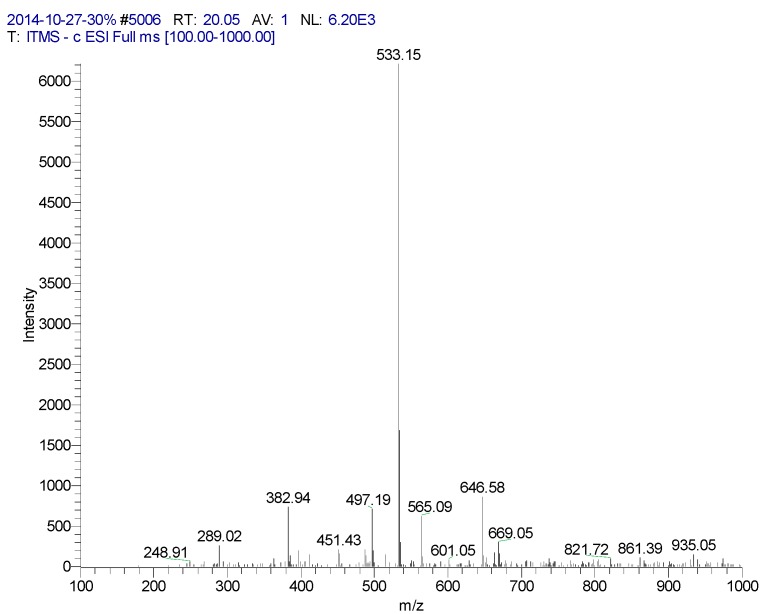
Mass spectrometry of CN30 extract by HPLC in the 20.05 min peak.

**Figure 8:**
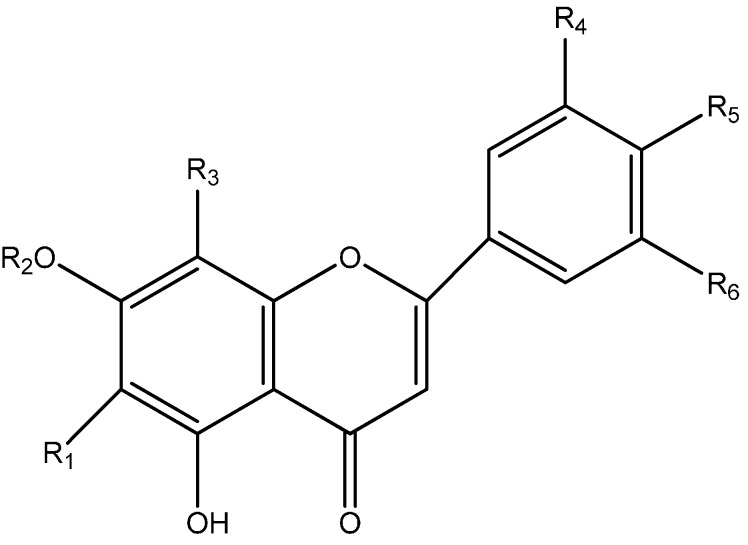
Proposed structure of flavonols glycosides found in CN30.

**Figure 9:**
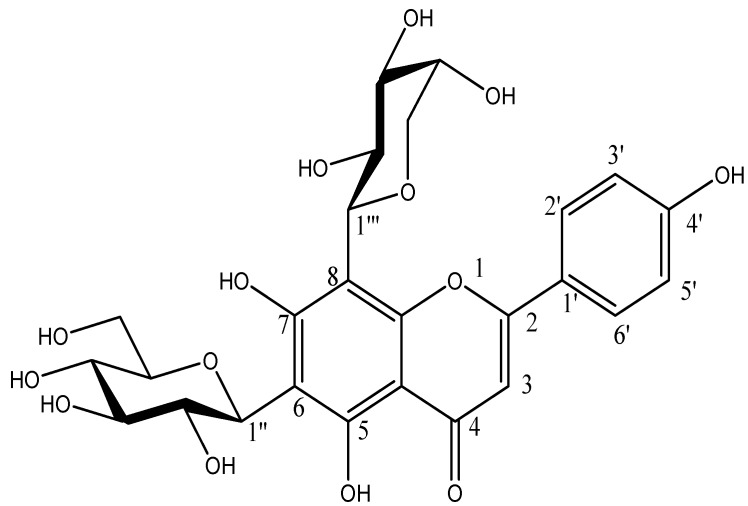
Structure of apigenin 6-*C*-β-d-glucopyranosyl-8-*C*-α-l-arabinopyranoside.

**Figure 10:**
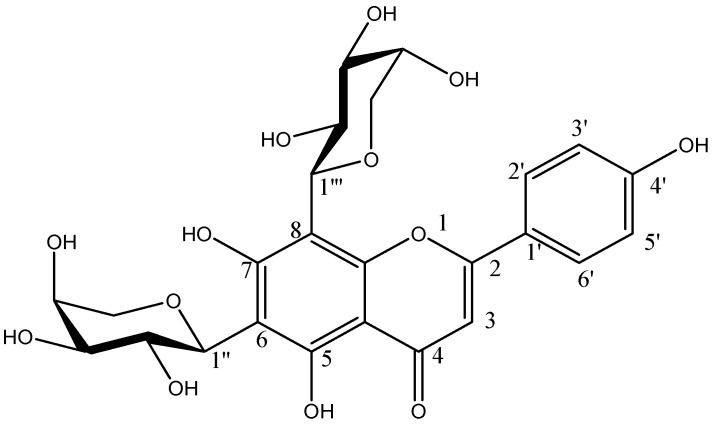
Structure of apigenin 6,8-di-*C*-α-l-arabinopyranoside.

The chemical profile of a 30% ethanol extract of *C. nutans* (CN30) was shown in Figure 1 and identified by HPLC and LC-MS based on the HPLC fingerprints, molecular ions [M − H]^−^, and MS/MS fragment ions analysis. CN30 contained seven compounds that were identified based on the HPLC fingerprints, molecular ions [M − H]^−^, and MS/MS fragment ions analyses. The identified compounds were shaftoside, orientin, vitexin, isoorientin, isovitexin and 6,8-apigenin-*C*-α-l-pyranarabinoside (refer to Figure 1, Figure 2, Figure 3, Figure 4, Figure 5, Figure 6, Figure 7, Figure 8, Figure 9 and Figure 10). The identities of these molecules were additionally confirmed with the aid of ^1^H-NMR analysis, which was consistent with previously reported data [11].

Analytical HPLC was used to determine the purities of the seven major compounds obtained from CN30 by HSCCC (shown in Figure 2). The HPLC analysis of each fraction, which revealed that the components eluted in the order of peaks, was performed by MS, ^1^H-NMR, and ^13^C-NMR analysis, as follows. These phytochemicals are the active constituents of CN30 showing antitumor effects [12,13,14,15,16,17].

To identify the active components contributing to the up-regulation of the immune response (UIR) efficacy of *C. nutans*, a bioassay-guided fractionation and purification process was performed to elucidate its bioactive fractions and compounds.

A total of seven compounds were isolated from the ethanol extract of the aerial parts of CN30. The known compounds (four) were identified by comparison of their physical and spectroscopic data to those reported in the literature (Table 1 and Figure 2).

**Table 1:** High-performance liquid chromatography-mass spectrometry (HPLC/MS) data, protonated and deprotonated molecules (*m*/*z*) for peaks, including the retention times (*R_t_*), MS/MS experiments, and maximal absorption wavelength (λ_max_) of the constituents found in *C. nutans.*

*R_t_* (min)	Tentative Compound	[M – H]^−^ (*m*/*z*)	Fragment Ions, (*m*/*z*)	Fragment Ions of [M − H]^−^
14.11	Isoorientin	579	288, 383, 563, 579, 692	235, 270 (99.14), 346 (100), 395,
14.37	Orientin	579	288, 382, 579, 692, 714	239, 258 (97.22), 348 (100), 349 (100), 440
16.33	Isovitexin	563	244, 289, 382, 447, 549, 563, 676	242 (96.17), 258 (100), 296, 321, 329, 338
17.60	Vitexin	533	533	235, 270 (99.14), 347 (100)
18.66	Apigenin6-C-β-d-glucopyranosyl-8-C-α-l-arabinopyranoside	563	249, 289, 335, 382, 397, 447, 512, 563, 676	234, 272 (98.95), 335 (100), 396
20.05	6,8-Apigenin-C-α-l-pyranarabinoside	533	289, 382, 497, 533, 565, 646	235, 271, 335 (100), 396

The optimized and validated method was applied to determine the concentration of the compounds in CN30 including Shaftoside (Apigenin6-*C*-β-d-glucopyranosyl-8-*C*-α-l-arabinopyranoside), Apigenin 6,8-C-α-l-pyranarabinoside, orientin, isoorientin, isovitexin and vitexin. The structures of the new compounds (three) were established by interpretation of their spectroscopic data, in particular by 2D MS. Proposed structure of flavonol glycosides found in CN30 are shown in Figure 8 and Table 2.

**Table 2:** Proposed structure of flavonols glycosides found in CN30.

Proposed Structure	R_1_	R_2_	R_3_	R_4_	R_5_	R_6_
Shaftoside	Glc	H	Rha	H	OH	H
Isoorientin	Glc	H	H	H	OH	H
Orientin	H	H	Glc	H	OH	OH
Isovitexin	Glc-	H	H	H	OH	H
Vitexin	H	H	Glc	H	OH	H
Apigenin 6,8-di-*C*-α-l-arabinopyranoside	Rha	H	Rha	H	OH	H

### 2.2. Identification of Compounds

Flavonoids were identified by LC-MS, ^1^H- and ^13^C-NMR, and direct TLC and PLC comparisons with authentic samples. TLC, HPLC, LC-MS, and ^1^H- and ^13^C-NMR data of the isolated flavonoids are as follows.

*Compound_CN30_* **1**: colorless needles (methanol), mp 235–237 °C, ESI-MS *m*/*z*: 168.0 [M − H]^−^. ^1^H-NMR (400 MHz, DMSO) δ 12.25 (s, 3H), 9.21 (s, 6H), 8.85 (s, 3H), 6.91 (s, 6H), 3.37 (d, *J* = 4.7 Hz, 16H), 2.55–2.46 (m, 5H). ^13^C-NMR (100 MHz, CD_3_OD) δ: 170.4 (-COOH), 146.4 (C-3, 5), 139.6(C-4), 122.2 (C-1), 110.5 (C-2, 6). Compound **1** by NMR spectral data: it can be concluded that the compound was gallic acid. After the gallic acid known reference co-thin detection, *R_f_* values are the same and the color is exactly the same, so this compound has been identified as gallic acid.

*Compound_CN30_* **2**: yellow powder, HPLC: (*R_t_* = 14.11 min). LC-MS: 447 [M − H]^−^, 895 [2M − H]^−^, 471 [M + Na]^+^, 919 [2M + Na]^+^, 449 [M + H]^+^, 487 [M + K]^+^.Calc. for C_21_H_20_O_11_. ^1^H-NMR (500 MHz, DMSO-*d*_6_) δ: 13.55 (1H, brs, 5-OH), 7.44 (1H, dd, *J* = 2.5 Hz, 9.0 Hz, 6′-H), 7.38 (1H, d, *J* = 2.5 Hz, 2′-H), 6.90 (1H, d, *J* = 9.0 Hz, 5′-H), 6.64 (1H, S, 3-H), 4.58 (1H, d, *J* = 10.0 Hz, 1′′-H). ^13^C-NMR (500 MHz, DMSO-*d*_6_) δ: 163.44 (C-2), 102.38 (C-3), 181.45 (C-4), 160.59 (C-5), 108.88 (C-6), 163.44 (C-7), 93.73 (C-8), 156.27 (C-9), 102.79 (C-10), 121.56 (C-1′), 112.92 (C-2′), 145.95 (C-3′), 150.44 (C-4′), 116.00 (C-5′), 118.82 (C-6′), 73.18 (C-1′′), 70.50 (C-2′′), 78.95 (C-3′′), 70.19 (C-4′′), 81.35 (C-5′′), 61.34 (C-6′′). Compound **2** was finally identified as isoorientin by comparing with these NMR spectral data and after isoorientin known reference co-thin detection, *R_f_* values are the same. The color is exactly the same.

*Compound_CN30_* **3**: yellow powder, HPLC: (*R**_t_*** = 14.37 min), ES-MS: [M – H]^−^447 *m*/*z*. calc. for C_21_H_20_O_11_. ^1^H-NMR (500MHz, DMSO-*d*_6_) δ: 3.22–3.88 (6H, m, glucosyl-H), 4.72 (1H,d, *J* = 9.0 Hz, H-1′′), 6.25 (1H, s, H-6), 6.65 (1H, s, H-3), 6.90 (1H, d, *J* = 8.2 Hz, H-5′), 7.44 (1H, d, *J* = 2.1 Hz, 2′-H), 7.50(1H, dd, *J* = 2.1, 8.0Hz, H-6′), 13.15 (1H, s, 5-OH). ^13^C-NMR (125 MHz, DMSO-*d_6_*) δ: 164.16 (C-2), 102.41 (C-3), 182.03 (C-4), 160.47 (C-5), 98.31 (C-6), 162.80 (C-7), 104.65 (C-8), 156.01 (C-9), 103.79 (C-10), 121.97 (C-1′), 114.07 (C-2′), 145.95 (C-3′), 149.90 (C-4′), 115.78 (C-5′), 119.45 (C-6′), 73.50 (C-1′′), 70.90 (C-2′′), 78.88 (C-3′′), 70.83 (C-4′′), 82.04 (C-5′′), 61.76 (C-6′′). Compound **3** was finally identified as orientin by comparing with these NMR spectral data and after orientin known reference co-thins detection, *R_f_* values are the same. The color is exactly the same.

*Compound_CN30_* **4**: yellow powder, HPLC: (*R_t_* = 16.33min), TOF-MS: 455 [M + Na]^+^, 432 [M − H]^−^, 471 [M + K]^+^, 887 [2M + Na]^+^, 903 [2M + K]^+^ calc for C_21_H_20_O_10_. ^1^H-NMR (500 MHz, DMSO-*d*_6_) δ: 13.46 (1H, brs, 5-OH), 7.85 (2H, d, *J* = 8.5 Hz, 3′,5′-H), 6.94 (2H, d, *J* = 8.4 Hz, 2′,6′-H), 6.71 (1H, s, 3-H), 6.42 (1H, s, 8-H), 4.56 (1H, d, *J* = 9.8 Hz, 1′′-H). ^13^C-NMR (500 MHz, DMSO-*d*_6_) δ: 163.32 (C-2), 102.60(C-3), 181.73 (C-4), 160.64 (C-5), 108.95 (C-6), 163.32 (C-7), 93.79 (C-8), 156.31 (C-9), 102.95 (C-10), 121.00 (C-1′), 128.35 (C-2′, 6′), 116.01 (C-3′, 5)′, 161.32 (C-′4), 73.13 (C-1′′), 70.52 (C-2′′), 78.93 (C-3′′), 70.20 (C-4′′), 81.38 (C-5′′), 61.37 (C-6′′). Compound **4** was finally identified as isovitexin by comparing with these NMR spectral data and after isovitexin known reference co-thin detection, *R_f_* values are the same and the color is exactly the same.

*Compound_CN30_* **5**: HPLC: (*R_t_* = 17.60 min).ES-MS: 432 [M − H]^−^. calc. for C_21_H_20_O_10_. ^1^H-NMR (500 MHz, DMSO-*d*_6_) δ:4.94 (1H, d, *J* = 9.8 Hz, H-1′′) 6.44 (1H, s, H-6), 6.94 (1H, s, H-3), 7.05 (2H, d, *J* = 8.7 Hz, H-3′, 5′), 8.26 (2H, d, *J* = 8.7 Hz, 2′,6′-H), 10.35 (1H, s, 4-OH), 10.83 (1H, s, 7-OH), 13.17 (1H, s, 5-OH). ^13^C-NMR (125 MHz, DMSO-*d*_6_) δ: 164.98 (C-2), 102.51 (C-3), 182.73 (C-4), 155.64 (C-5), 98.45 (C-6), 162.31 (C-7), 104.56 (C-8), 160.28 (C-9), 104.07 (C-10), 122.07 (C-1′), 128.99 (C-2′,6′), 115.01 (C-3′,5′), 161.32 (C-4′), 73.93 (C-1′′), 71.03 (C-2′′), 79.01 (C-3′′), 70.20 (C-4′′), 81.29 (C-5′′), 61.36 (C-6′′). Compound **5** was finally identified as vitexin by comparing with these NMR spectral data and after vitexin known reference co-thin detection, *R_f_* values are the same and the color is exactly the same.

*Compound_CN30_* **6**: HPLC: (*R_t_* = 18.66 min), as a pale yellow powder, sprayed aluminum chloride reagent and yellow spots, ammonia fuming bright yellow spots, molish reaction was positive, indicating that the compound is a flavonoid glycoside. TOF MS ES + (*m*/*z*) 565, 587, 603 given spectral peaks, indicating that the compound has a molecular weight of 563 to [M − H]^−^ peak, [M + Na]^+^ peak of 587, 603 [M + K]^+^ peak. C_26_H_28_O_14_. ^1^H-NMR (400 MHz, DMSO-*d*_6_) spectrum: δ 13.90 (s, 1H) 5-OH flavone active hydrogen signals; δ 8.091 (brs, 2H) of the flavone ring B 2′,6′ bit signal hydrogen, δ 6.919 (2H, d, *J* = 8.8 Hz) of the B-ring flavonoid 3′,5′-hydrogen signal, as AA′BB′ coupling system, δ 6.720 (s, 1H) which presumably is the C-ring flavonoid 3 hydrogen signal-position. Shift value between 4.8 and 3.0 for the sugar hydrogen signal, δ 4.788 and δ 4.727 for the end of two protons on the sugar.

^13^C-NMR (400 MHz, DMSO-*d*_6_) spectrum of the low-field, the emergence of four carbon signals, respectively, between 163.607, 161.133, 159.678, 154.644. 80–60 ppm of carbon signal on sugar, occurs within the range 11 signal. We initially speculated that the flavonoid contains a five-carbon sugar and a six-carbon sugar. In addition, based on molecular weight, we found *Compound_CN30_* **6** is relatively larger than *Compound_CN30_* **7**, and difference between both two compounds was 30. With reference to Hu and colleagues [18], we may imply that skeleton structure of the *Compound_CN30_* **6** to be apigenin. Thus, we may consider that a low-field signal oxygenated carbon nucleus overlapped. Similarly, signals of carbon 6 and 8 of apigenin nucleus does not appear at 100–90 ppm, but moved to displaced downfield, so according to the general law of carbon flavonoid glycoside bond, we may hypothesize carbon 6 and 8 was connected with sugar by carbon-to-carbon bond and, this compound is 6,8-biglycoside apigenin. According to obtained HMQC spectrum, δ_C_ 75.211 and δ_H_ 4.788 are interlinked, which carbon and hydrogen signals are belonging to upper group in the arabinose. At the same time, δ_C_ 73.788 interacts with δ_H_ 4.727, both are carbon and hydrogen signals in glucose upper group. Based on HMBC spectrum, we can recognize that glucose is connected to carbon 6 and, arabinose to carbon 8.

According to ^13^C-NMR and ^1^H-NMR data reported in the literature that are basically the same [19], the compound is determined to be apigenin-6-*C*-β-d-glucose-8-*C*-α-l arabinose, *i.e.*, summer Buddha Tower glycosides.

For *Compound_CN30_* **6** during the drying process, acetic acid is not completely removed, the performance of the ^1^H-NMR δ of about 1.9 molecules of hydrogen as acetic signal is a sharp singlet, ^13^C-NMR in, δ 21 molecules of methyl acetate peak, in the HMQC spectrum, both associated compound ^13^C-NMR and ^1^H-NMR with the literature on the *Compound_CN30_* **6** as shown in Figure 9 and Table 3. The glucose 3, 4, 5-position hydrogen chemical shifts between δ 3.2–3.4, overlapping with the water peak, ^1^H-NMR is not marked, so there is no attribution for them.

**Table 3:** ^1^H-NMR & ^13^C-NMR data of compounds **6** in CN30.

Carbon	^13^C-NMR	^1^H-NMR	^13^C-NMR [19]	^1^H-NMR [19] (Reference Volume)
2	164.127		164.1	
3	102.333	6.774 (1H, s)	102.2	6.78 (1H, s)
4	182.308		182.3	
5	158.892		158.7	
6	108.588		108.4	
7	162.014		161.2	
8	104.786		104.7	
9	154.910		155.0	
10	103.427		103.5	
1′	121.331		121.1	
2′	129.360	8.149 (2H, brs)	129.4	8.15 (2H, brs)
3′	116.117	6.914, 6.892 (2H, d, *J* = 8.8 Hz)	116.0	6.91 (2H, d, *J* = 8.8 Hz)
4′	161.239		161.2	
5′	116.117	6.914,6.892 (2H, d, *J* = 8.8 Hz)	116.0	6.91 (2H, d, *J* = 8.8 Hz)
6′	129.360	8.149 (2H, brs)	129.4	8.15 (2H, brs)
6-Ara				
1′′	74.298	4.665 (1H, d, *J* = 9.2 Hz)	74.1	4.66 (1H, d, *J* = 9.5 Hz)
2′′	68.776	3.997 (1H, brm)	68.6	4.00 (1H, brm)
3′′	74.829	3.448 (1H, m)	74.6	3.44 (1H, m)
4''	69.126	3.802 (1H, m)	69.0	3.79 (1H,m)
5''	70.294	3.870, 3.606 (2 × 1H, 2 × m)	70.2	3.83, 3.60 (2 × 1H, 2 × m)
8-Ara				
1′′′	74.298	4.724 (1H ,d, *J* = 9.2 Hz)	74.1	4.72 (1H, d, *J* = 9.4 Hz)
2′′′	69.126	4.211 (1H, brm)	69.0	4.22 (1H, brm)
3′′′	75.031	3.487 (1H, m)	74.9	3.48 (1H, m)
4′′′	70.294	3.802 (1H, m)	70.2	3.85 (1H, m)
5′′′	71.059	3.901, 3.635 (2 × 1H, 2 × m)	71.0	3.90, 3.62 (2 × 1H, 2 × m)
5-OH		13.752 (1H, brs)		13.76 (1H, brs)

*Compound_CN30_* **7**: HPLC: (*R_t_* = 20.05 min) as a pale yellow powder, sprayed aluminum chloride reagent and yellow spots, ammonia fuming bright yellow spots, mulish reaction is positive, indicating that this compound is a flavonoid glycoside. TOF-MS-ES^+^ (*m*/*z*) spectrum gives peaks 533 for the [M − H]^−^ peak, [M + Na]^+^ peak of 557, 573 for the [M + K]^+^ peak indicating the molecular weight of the compound is 534.

^1^H-NMR (400 MHz, DMSO-*d*_6_) spectrum: δ 13.752 (s, 1H) 5-OH flavone active hydrogen signals; δ 8.149 (2H, brs) of the flavone ring B 2′,6′ bit signal hydrogen, δ 6.93 (d, 2H, *J* = 8.8 Hz) 3′,5′ hydrogen signal, as AA′BB′ coupling system, δ 6.83 (s, 1H); we speculated that flavonoids may be on the C-ring 3-hydrogen signal. Shift value between 3.0–4.8 for the sugar hydrogen signal, δ 4.724 and δ 4.665 for the end of two protons on the sugar.

Based on the ^13^C-NMR (400 MHz, DMSO-*d*_6_) spectrum of the low-field, we identified five peaks of 164.127, 162.014, 161.239, 158.892, 154.910, which indicated the compound contains three oxygen-carbon signals, and it can be implied that the molecule of the compound contains three hydroxyl groups. When comparing with the literature [19] alignments, it can be hypothesized that three OH-groups are connected to the 5, 7, 4′ position which supports, again, that the nucleus of the flavonoid is apigenin. However, in 100–90 ppm, carbon 6 and 8 signals of apigenin does not appear on the nucleus, but is shifted to downfield. Furthermore, based on the law of carbon flavonoid glycoside bonds, we also imply that carbons 6 and 8 are connected with the sugar by carbon-to-carbon bonds and the identified compound is 6,8-biglycoside apigenin. The signal between 80–60 ppm was typical of sugar carbon signals; only seven carbon signals can be detected, which may be resulted from overlapped carbon signals.

According to ^13^C-NMR and ^1^H-NMR data reported in the literature are essentially the same [20], it is determined that the compound is 6,8-apigenin-*C*-α-l-pyranarabinoside.

For *Compound_CN30_* **7**, during the drying process, acetic acid is not completely removed; the performance of the ^1^H-NMR δ of about 1.9 molecules of hydrogen as the acetic signal is a sharp singlet, ^13^C-NMR in, δ 21 molecules of methyl acetate peak, in the HMQC spectrum, both associated Compound ^13^C-NMR and ^1^H-NMR with the literature on *Compound_CN30_* **7** as shown in Figure 10 and Table 4.

These molecules confirmed by using NMR were in accordance with previously reported results. Interestingly, these seven phytochemicals present in CN30 have been reported as the active constituents of showing antitumor effects [12,13,14,15,16,17]. Hence, the following studies aimed to evaluate the inhibitory effect of CN30 on hepatoma *in vivo* and elucidate its possible mechanism.

**Table 4:** ^1^H-NMR & ^13^C-NMR data of compounds **7** in CN30.

Carbon	^13^C-NMR	^1^H-NMR	^13^C-NMR [19]	^1^H-NMR [1] (Reference Volume)
2	164.127		164.1	
3	102.333	6.774 (1H, s)	102.2	6.78 (1H, s)
4	182.308		182.3	
5	158.892		158.7	
6	108.588		108.4	
7	162.014		161.2	
8	104.786		104.7	
9	154.910		155.0	
10	103.427		103.5	
1′	121.331		121.1	
2′	129.360	8.149 (2H, brs)	129.4	8.15 (2H, brs)
3′	116.117	6.914, 6.892 (2H, d, *J* = 8.8 Hz)	116.0	6.91 (2H, d, *J* = 8.8 Hz)
4′	161.239		161.2	
5′	116.117	6.914, 6.892 (2H, d, *J* = 8.8 Hz)	116.0	6.91 (2H, d, *J* = 8.8 Hz)
6′	129.360	8.149 (2H, brs)	129.4	8.15 (2H, brs)
6-Ara				
1′′	74.298	4.665 (1H, d, *J* = 9.2 Hz)	74.1	4.66 (1H, d, *J* = 9.5 Hz)
2′′	68.776	3.997 (1H, brm)	68.6	4.00 (1H, brm)
3′′	74.829	3.448 (1H, m)	74.6	3.44 (1H,m)
4′′	69.126	3.802 (1H, m)	69.0	3.79 (1H, m)
5''	70.294	3.870, 3.606 (2 × 1H, 2 × m)	70.2	3.83, 3.60(2 × 1H, 2 × m)
8-Ara				
1′′′	74.298	4.724 (1H, d, *J* = 9.2 Hz)	74.1	4.72 (1H, d, *J* = 9.4 Hz)
2′′′	69.126	4.211 (1H, brm)	69.0	4.22 (1H, brm)
3′′′	75.031	3.487 (1H, m)	74.9	3.48 (1H, m)
4′′′	70.294	3.802 (1H, m)	70.2	3.85 (1H, m)
5′′′	71.059	3.901, 3.635 (2 × 1H, 2 × m)	71.0	3.90, 3.62(2 × 1H, 2 × m)
5-OH		13.752 (1H, brs)		13.76 (1H, brs)

### 2.3. Effect of CN30 on HepAXenograf Growth in Vivo

**Figure 11:**
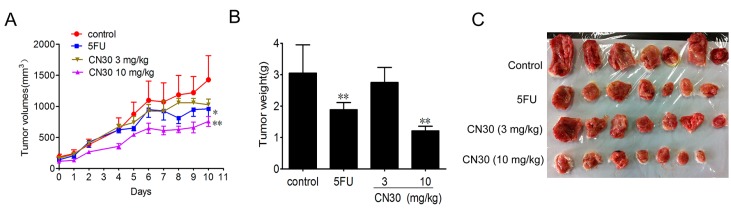
CN30 inhibited HepAxenograft growth *in vivo.* HepA tumor cells (1 × 10^7^; grown in donor mice) were transplanted subcutaneously into the axilla of the ICR mice. Seventy-two hours after tumor cell transplantation, mice were randomly allocated to control and treatment groups according to tumor size, with 10 mice per group. Treatments were administered on days 0–10. Tumor volume was measured every day (**A**). After the mice were sacrificed, solid tumors were separated. Dissected tumors coming from control (vehicle-treated) mice, fluorouracil-treated (20 mg/kg) mice, and CN30-treated (3 or 10 mg/kg) mice were weighed (**B**) and photographed (**C**). Results in (**A**) and (**B**) were shown as mean ± SD and a significant difference between treatment and control groups was indicated by * *p* < 0.05, ** *p*< 0.01.

As indicated in Figure 11, CN30 treatment led to a significant and dose-dependent reduction in tumor size and weight with the inhibition ratios of 8.2% and 58.6% at doses of 3 and 10 mg/kg, respectively (*p* < 0.05). Specifically, fluorouracil also reduced 37.1% of tumor weight, which was significantly lower than that treated with 10 mg/kg of CN30 (*p* < 0.05).

**Figure 12:**
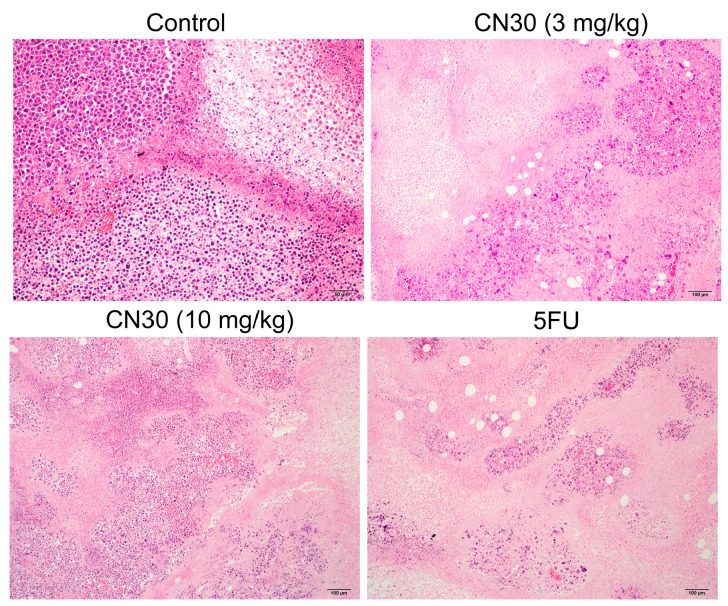
Hematoxylin-eosin (H & E) staining of tumor tissue Paraffin sections of tumor tissues from mice were analyzed by H & E staining. Representative images are shown for each group (Scale bar for Control Group represents 50 μm, while the other 3 Groups represent 100 μm).

Hematoxylin and eosin (H & E) staining was used to verify the antitumor activity of CN30. From Figure 12, a different cellular architecture and typical pathological characteristics of malignancy were observed in the corresponding specimens from mice with HepAxenografts treated by CN30 or fluorouracil. CN30 (10 mg/kg) and fluorouracil had the similar effect on the tumor cell damages, such as condensation of cytoplasm and pyknosis of nuclei. Hence, it could be concluded that CN30 reduced the tumor volume and growth rate of HepA cells *in vivo*.

### 2.4. Effect of CN30 Treatment on Body Weight and Immune Organ Index

**Figure 13:**
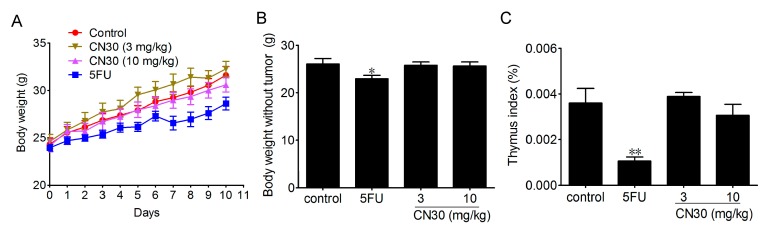
Effects of CN30 treatment on body weight, body weight without tumor, and thymus index of tumor-bearing mice. (**A**) Body weight changes in tumor-bearing mice in each group. (**B**) Bodyweight without tumor in each group. (**C**) Thymus index of tumor-bearing mice. Results were shown as mean ± SD (*n* = 10) and a significant difference between treatment and control groups was indicated by * *p* < 0.05, ** *p* < 0.01.

Thymus index is closely related to immune function. In tumor-bearing mice, these two indices were often used for the evaluation of immune function. The body weight and thymus index of experimental mice treated with CN30 (25.8 ± 1.8 g for CN30 3 mg/kg group and 25.7 ± 1.9 g for CN30 10 mg/kg group) and fluorouracil (control) were as shown in Figure 13. The body weights of mice treated with CN30 were found to be significantly higher than those treated with fluorouracil (22.9 ± 1.9 g). The thymus was almost diminished in fluorouracil treated mice (thymus index: 0.00106 ± 0.00044) while CN30 has no effect on thymus (thymus index: 0.00307 ± 0.00017) compared with normal mice (thymus index: 0.0036 ± 0.00016).

### 2.5. Effect of CN30 on Induced Tumor Cell Apoptosis

**Figure 14:**
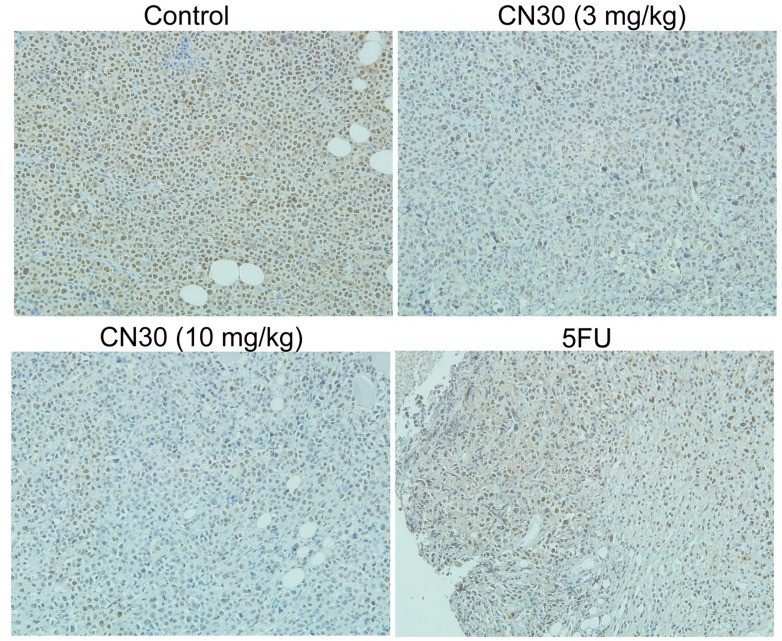
Immunohistochemistry analysis of proliferating cell nuclear antigen (PCNA) in tumor tissues. Paraffin sections of tumor tissues from mice were analyzed by immunehistochemical staining. Representative images show PCNA expression (magnification, ×200) in tumors. (Scale bar for Control Group represents 50 μm, while the other 3 Groups represent 100 μm).

**Figure 15:**
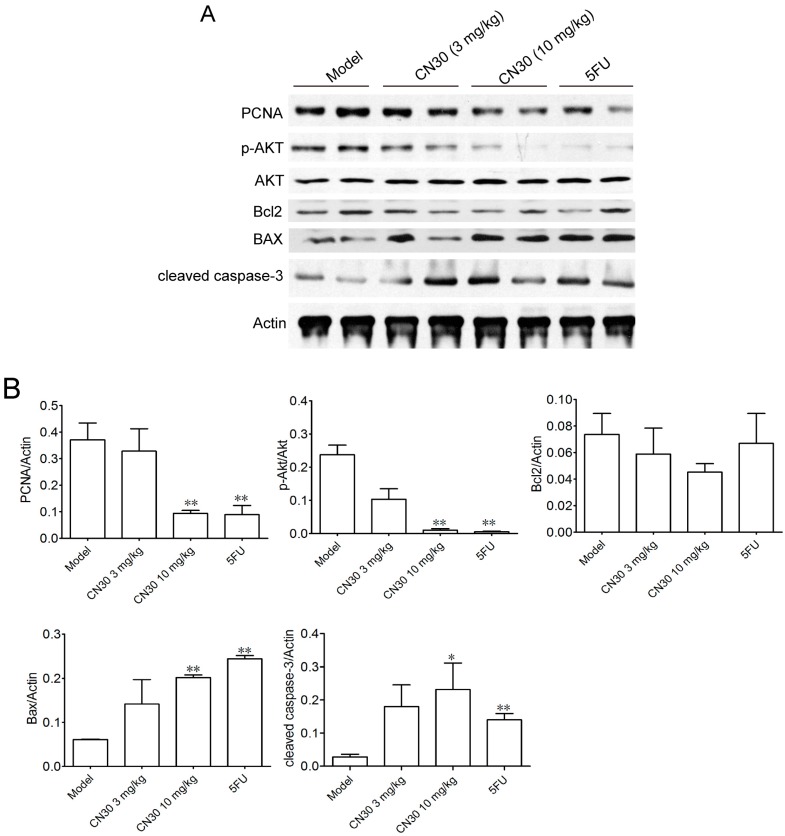
(**A**) Western blots of apoptosis-related protein in tumor tissues. Protein of tumor tissue from mice in each group were extracted and analyzed by western blot. PCNA, *p*-AKT, Bcl2, BAX and caspase-3 in tumor tissues were examined. Actin was used as the loading control. (**B**) PCNA, *p*-AKT, Bcl2, BAX and caspase-3 in tumor tissues were shown as mean ± SD (*n* = 10) and a significant difference between treatment and control groups was indicated by *p < 0.05, ** *p* < 0.01.

As shown in Figure 14, immunohistochemical staining and Western blot indicated that the expression of proliferating cell nuclear antigen (PCNA) was significantly decreased after CN30 treatment (Figure 14 and Figure 15). Treatment with CN30 caused cleavage ofcaspase-3, suggesting the initiation of the apoptosis pathway. Likewise, the expression level of the pro-apoptotic protein BAX was increased, with a corresponding decrease in the anti-apoptotic protein Bcl2 in treated tumor-bearing models suggestive of induced apoptosis by a shift in the BAX: Bcl2 ratio favoring apoptosis. Furthermore, the expression of *p*-AKT, cleaved-caspase-3, and BAX was significantly increased in treated models.

Proteins from tumor tissues from mice in each group were extracted and analyzed by Western blot. PCNA, *p*-AKT, Bcl2, BAX, and caspase-3 were examined in tumor tissues. Actin was used as the loading control.

### 2.6. CN30 Treatment Enhanced CD8^+^ T Cell Infiltration

**Figure 16:**
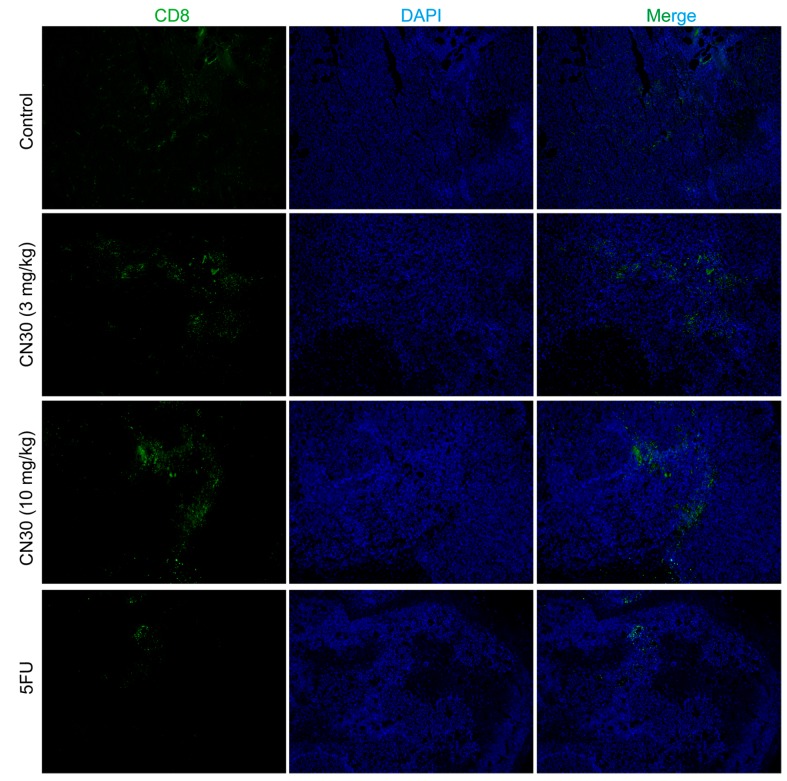
Immunofluorescence analysis of CD8^+^ cell infiltration in the tumor tissues in each group. Sections of tumor tissue from each group were stained with CD8^+^-FITC (Fluorescein isothiocyanate), DAPI (4′,6-diamidino-2-phenylindole) and photographed by fluorescence microscopy (×100). (Scale bar for Control Group represents 50 μm, while the other 3 Groups represent 100 μm).

CD8^+^ T cells have been reported to play an important role in antitumor immunity. As shown in Figure 16, treatment with CN30 significantly enhanced the infiltration of CD8^+^ T cells to HepA tumor cells compared with the untreated control group, indicating that CN30 treatment could promote the function of CD8^+^ T cells.

### 2.7. Effect of CN30 on Serum Cytokines Levels in HepA-Bearing Mice

**Figure 17:**
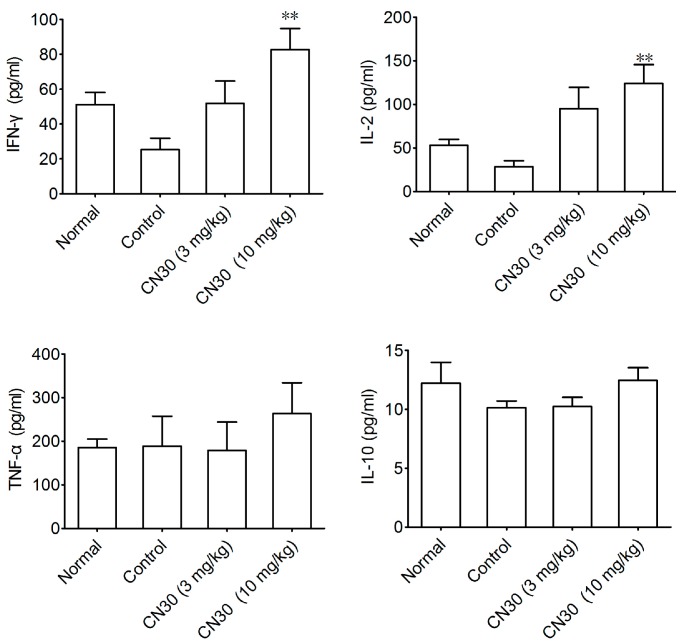
Effect of CN30 treatment on IFN-γ, IL-2, TNF-α, and IL-10 levels in the serum of tumor-bearing mice. The serum of the mice in each group was taken and cytokine levels were analyzed by enzyme-linked immunosorbent assay (ELISA). Data are presented as mean ± SD (*n* = 10). ** *p* < 0.01 *vs.* control group.

We investigated the effect of CN30 on IFN-γ and IL-2, TNF-α, and IL-10 levels in HepA tumor-bearing mice. As seen in Figure 17, compared with those of the control group, IFN-γ and IL-2 levels of mice treated with CN30 were significantly increased (IFN-γ: control group 25.4 ± 4.8 pg/mL, CN30 10 mg/kg group 82.8 ± 10.2 pg/mL. IL-2: control group 29.3 ± 6.8 pg/mL, CN30 10 mg/kg group 112.2 ± 14.1 pg/mL), while TNF-α and IL-10 showed no obvious change.

### 2.8. CN30 Treatment Promoted Th1 Cell Differentiation in HepA-Bearing Mice

**Figure 18:**
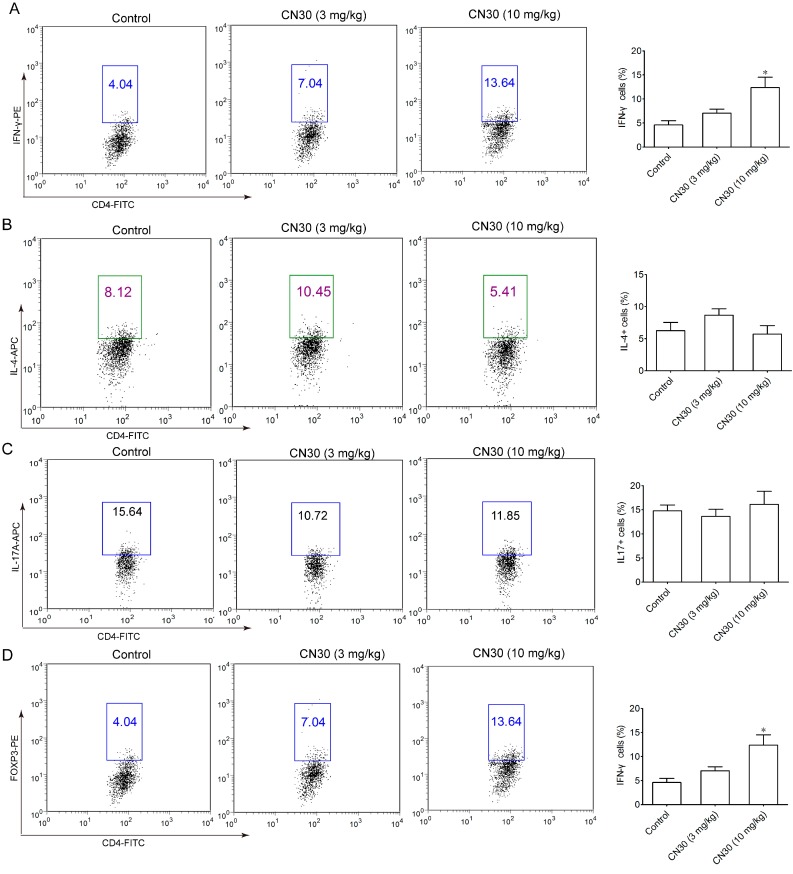
Intracellular staining of Th1 cells in the spleen of tumor-bearing mice. CN30 could significantly elevate the ratio of IFN-γ^+^ CD4^+^ T cells (Th1) in treated tumor-bearing mice (**A**) (control group 4.6%, CN30 10 mg/kg group 12.4%) while levels of IL-4^+^ CD4^+^ T cells (Th2) were decreased slightly (control group 6.3%, CN30 10 mg/kg group 5.7%) (**B**). Levels of IL-17A^+^ CD4^+^ T cells and FOXP3^+^ CD4^+^ T cells remained unchanged (**C**,**D**).

The effect of CN30 on T helper cells was investigated by analyzing the levels of T cell subsets in the spleen of tumor-bearing mice by intracellular staining (Figure 18). Spleen T cells from each group were extracted and stimulated by phorbol 12-myristate 13-acetate (PMA)/ionomycin/monensin for 6 h. The subsets were examined by intracellular staining. Data are presented as mean ± SD (*n* = 3). * *p* < 0.05 *vs.* control group.

### 2.9. Discussion

HCC is the most common type of liver cancer and is the fifth most common malignancy worldwide, with more than 500,000 new cases of HCC annually [20,21]. The most commonly available treatment options for HCC are orthotopic liver transplantation, surgical resection, local destruction, and chemotherapy. However, the level of success of such treatment for tumors is largely dependent on the size and location of the tumor at the time of diagnosis, as well as the side effects and toxicities associated with most of the approved drugs for HCC treatment [22,23]. As such, there has been an extensive search for drugs that are effective in the prevention and management of HCC, and compounds with natural origins are attracting increasing interest and have the potential to become suitable options for the prevention and treatment of HCC.

*Clinacanthus nutan* is a well-known medicinal plant widely used in Thai traditional medicine, has been used for the treatment of HZV lesions, insect bites, snake bites, and hepatitis [24]. Our results from both HPLC and MS analysis indicated the presence of flavone c-glycosides as the active constituents in CN30 extracts.

Recent studies have demonstrated that the immune system plays a critical role in the antitumor defense and immunomodulation could be a promising strategy in targeting tumor cells [25]. The spleen and thymus indices play an important role in the functioning of the immune systems of different organisms. T cells are involved in the process of tumor initiation, development, and metastasis inhibition. Mature T cells mainly differentiate into CD4^+^ and CD8^+^ cells [26,27]. CD4^+^ T cells induce the maturation of B cells into plasma cells and the activation of cytotoxic T cells, including CD8^+^ cells [28]. With the help of activated CD4^+^ T cells, CD8^+^ T cells migrate to the tumor site and exert a specific cytotoxic effect. Perforin and granzymes are released from CD8^+^ T cells [29]. Perforin forms pores in the cell membrane of the target cell, creating an aqueous channel through which the granzymes enter, leading to cell apoptosis via degradation of target cell DNA or stimulation of the FasL/Fas pathway [30,31]. Our results indicated that the thymus index of tumor-bearing mice treated with CN30 was significantly enhanced compared with those of fluorouracil-treated and untreated HepA tumor-bearing mice, which had decreased thymus indices. Likewise, CD8^+^ T cells were largely presented in the tumor tissues after CN30 treatment, which suggests that CN30 has protective effects on immune function by possibly promoting the functions of CD8^+^ T cells in suppressing HepA tumor growth.

CD4^+^ T lymphocytes are classified mainly as Th1, Th2, and Th17 subsets, according to the type of cytokines secreted. However, these subsets play different roles during tumor development. Th1 cells mainly secrete IL-2 and IFN-γ, which can suppress tumor growth by promoting CTL function [32,33]. IL-2 is an important component of the cytokine network and is believed to promote lymphocyte mitosis, which enhances the osmotic lysis function of the killer cells and assists in generating antibodies. IFN-γ is an important cytokine that is critically involved in the innate immune response. It increases the expression of Class II MHC molecules and activation of macrophages. These cytokines are increased in cancer and tumor cells to boost the immune function. Th2 cells mainly secrete IL-4, and IL-10, which are involved in humoral immune and allergic reactions and have been shown to promote tumor growth by stimulating cell proliferation or conferring tumor cell resistance to apoptosis [34,35,36]. Tregs characterized by FOXP3 transcription factor may foster tumor expansion by suppressing CD8^+^ CTLs and NK cells [37]. Th17-derived cytokine IL-17 can stimulate STAT3 signaling in tumors or promote myeloid-derived suppressor cell-mediated tumor-promoting microenvironments [38,39]. In the present study, the levels of serum IL-2 and IFN-γ were significantly increased, while a corresponding decrease in serum levels of IL-4 was observed in CN30-treated tumor-bearing mice.

Thus, the aforementioned results demonstrated that CN30 exerted its antitumor activity by enhancement of immune cytokine levels in the serum, thereby promoting the immune response in HepA tumor-bearing mice. These findings provide evidence for the application of *C. Nutans* in the treatment of cancers.

## 3. Experimental Section

### 3.1. Chemicals

Fluorouracil was purchased from Nantong Jinghua PharmaceuticalCo., Ltd (Nantong, Jiangsu, China). Antibodies against *p*-AKT, caspase-3, BAX, and Bcl-2were procured from Cell Signaling Technology (Danvers, MA, USA). PCNA and β-Actin were obtained from Santa Cruz Biotechnology Inc. (Dallas, TX, USA). Hematoxylin and eosin dye were purchased from Nanjing Jiancheng Bioengineering Institute (Nanjing, Jiangsu, China). RIPM1640 medium and fetal bovine serum (FBS) were obtained from Life Technology (Grand Island, NY, USA). ELISA assay kits for TNF-α, IFN-γ, IL-10, and IL-2 were purchased from Dakewe Biotech Company (Shenzhen, Guangdong, China). GTVisin™ anti-mouse/anti-rabbit immunohistochemical analysis kit was bought from Gene Company (Shanghai, China). FITC-anti-CD8, FITC-anti-CD4, PE-anti-IFN-γ, APC-anti-IL-17A, PE-anti-FOXP3, APC-anti-IL-4, and intracellular staining kits were purchased from eBioscience (San Diego, CA, USA). All other chemicals were obtained from Sigma-Aldrich (St. Louis, MO, USA).

### 3.2. Plant Material

Fresh aerial parts of *C. nutans* were collected in November 2014 from Seremban, Negeri Sembilan province, Malaysia. The specimens were authenticated by Professor Jun Chen at the School of Pharmacy, Jiangsu University, China. A voucher specimen was stored at the herbarium of Jiangsu University.

### 3.3. Extraction and Isolation

The dried aerial part of *C. nutans* (Burm. f) Lindau (2.0 kg) were first extracted twice with six-fold 95% ethanol for 2 h each at reflux, evaporated under reduced pressure to remove ethanol and concentrated to yield a viscous ethanol extract vacuum distillation extract, dissolved in water, and filtered. This extract was water-soluble (382 g yield 19.1%, *w*/*w*). The crude extracts were combined and subjected to further fractionation on a Diaion HP-20 macroporous adsorption resins (Mitsubishi Kasei, Tokyo, Japan) column (10 cm × 1500 cm). After reaching the adsorption equilibrium, resins were first eluted with deionized water and then consecutively eluted with 30%, 70%, and 90% ethanol solution. After concentration and freeze drying, the ethanol extract was further partitioned using the aqueous fraction (AF yield 49.2%, *w*/*w*), 30% ethanol fraction (CN30, yield 9.1%, *w*/*w*), 70% ethanol fraction (CN70, yield 18.7%, *w*/*w*), and 90% ethanol fraction (CN90, yield 19.6%, *w*/*w*), and were subjected to plant material for pharmacological study.

The guided isolation and purification of major components in active fractions were performed under monitoring by HPLC and TLP analysis, and major components were tracked by comparison of HPLC chromatograms of the active fraction and its sub-fractions.

The optimum conditions for thin layer chromatography were confirmed: 2 μL of the sample solution was applied to silica gel G plates. After detection, butanol–acetic acid–water (BAW) (*v*/*v*/*v*) 4:1:5 was used as the mobile phase. The flow rate was 0.4 mL/min. There appeared to be clear and concentrated spots in the iodine-cylinder.

Solutions in part by HP-20 column (100 × 600), first with water, then eluted with ethanol, evaporated to dryness via ethanol elution to give 10 g of brown extract (A), by means of Sephadex LH-20 (5%–30% ethanol gradient elution) to give two portions Min: B (1 g), C (3 g). The two parts are by MCI-gel (10%–50% methanol gradient elution) to give four components (I–IV), and several components were purified by silica gel (200–300 mesh) 100–200 mesh polyamide column, with H_2_O and 30% and 60% ethanol gradient elution, (*v*/*v*, 25:75, H_2_O–MeOH). Column chromatography, eluted with system CHCl_3_–MeOH–H_2_O (100:10:1–80:20:1), most after using ODS (eluting system: MeOH–H_2_O, 10% to 70%) to give compounds I (147 mg) and II (73 mg).

### 3.4. LC/MS/MS Experiment

A fresh petal was homogenized and extracted with the mobile phase consisting of (A) 0.1% formic acid in water and (B) methanol. The crude extract was suspended in H_2_O and passed through a 0.5 μm Fluorpore Millipore membrane from Merck Inc. (Darul Ehsan, Malaysia) prior to injection (10 μL aliquot) into the Cosmosil™ C18-AR-II, LC Column (150 mm × 4.6 mm, 5 μm), Nacalai HPLC system(Kyoto, Japan). The flavonoids in the eluates were analyzed with an LC/MS/MS system; the eluates from a Shimadzu GC 20 instrument (Kyoto, Japan) equipped with a C18-AR-II, LC Column and a Finngan surveyor PDA plus Detector was introduced into an Esquire3000 ion trap mass spectrometer, LXQ from Thermo Scientific (Waltham, MA, USA). MS data were collected in auto-MS/MS mode. The samples were eluted at 35 °C with a flow rate of 1.0 mL/min. Detection wavelength was 280 nm. The solvent system used was a linear gradient of 1% to 90% solvent D (methanol) in solvent C (0.1% formic acid) over a period of 45 min.

### 3.5. Animal Studies

All animal welfare and experimental procedures were carried out in strict accordance to the Guidelines for the Care and Use of Laboratory Animals (The Ministry of Science and Technology of China, 2006) and the related ethical regulations of the university. ICR mice (with normal T/B cells, 6–8 weeks, 18–22 g) were obtained from The Experimental Animal Center of Jiangsu University. HepAhepatocarcinoma tumor cells were obtained from Jiangsu Cancer Hospital and cultured in the aseptic form for tumor-bearing mice. Ascites were drawn from hepatocarcinoma mice (HepA) under aseptic conditions, and diluted in normal saline and subcutaneously implanted by injecting 0.2 mL of PBS containing 1 × 10^7^ viable tumor cells under the skin on the right oxter of the mice. After 72 h of implantation, the tumor-bearing mice were randomly assigned to one of four experimental groups (10 mice per group), according to tumor size. Group one was injected with the tumor cells but treated with water only, group two was injected with tumor cells and received intraperitoneal injections of fluorouracil (20 mg/kg), and groups three and four were injected with tumor cells and treated with CN30 (3 mg/kg and 10 mg/kg, respectively) for 10 consecutive days through a gastric probe. The mice were weighed every day. On the 10th day, mice were weighed and euthanized, and tumors were removed and the weight measured. Tumor volumes were measured and calculated using the equation volume = a × b_2_/2, where “a” is the maximal width and “b” is maximal orthogonal width. The tumor wet weights of the treated (*T_w_*) and control (*C_w_*) groups were measured on the last day of each experiment, and the percentage of tumor growth inhibition was calculated as follows: Inhibition (%) = [1− (*T_w_*/*C_w_*)] ×100%.

### 3.6. Hematoxylin-Eosin Staining

H & E staining was performed on sections that were cut from tumor specimens that had been fixed in 4% buffered formalin and embedded in paraffin. The stained tissues were examined under a light microscope.

### 3.7. Western Blot Analysis

Tissue lysates were prepared from xenograft tumors. Protein extracts were obtained and the protein concentration was determined using the Bradford Protein Assay Reagent. Equal amounts of total protein lysates were loaded on 10% SDS-PAGE and transferred onto PVDF membranes. After blocking, the membranes were incubated overnight with various primary antibodies at 4 °C. After washing three times with TBST, the blot was further incubated for 2 h at room temperature with horseradish peroxidase-conjugated secondary antibodies. After washing three times with TBST, target proteins were detected by the ECL system.

### 3.8. Immunofluorescence Histochemistry

CD8^+^ T cell infiltration analysis was performed on paraffin-embedded colonic tissue sections (5 μm). Briefly, the sections were deparaffinized in xylene, rehydrated through graded ethanol, and rinsed with distilled water and 1% PBS-Tween. Endogenous peroxidase activity was blocked using 2% hydrogen peroxide for 10 min. The sections were further incubated with 3% goat serum to block non-specific protein staining. The sections were further incubated for 2 h at room temperature with primary antibodies anti-CD8-FITC (1:100) and then overnight at 4 °C. The slides were then counter-stained with DAPI for 2 min. The reaction was stopped by thorough washing with water for 20 min. Images were visualized by confocal laser-scanning microscope (Olympus, Lake Success, NY, USA).

### 3.9. Determination of Biochemical Parameters

Blood samples were collected from tumor-bearing mice prior to sacrifice by cervical dislocation, deposited for 1 h at 4 °C, and centrifuged at 3000 *g* for 15 min. Subsequently, the serum was collected for the determination of IL-17A, IL-10, IFN-γ and TNF-α level according to the protocols available in the commercially available ELISA kit.

### 3.10. Intracellular Staining

The intracellular expression of IL-17A, IL-4, FOXP3 and IFN-γin CD4^+^ T cells was analyzed using an eBioscienceintracellular staining kit (San Diego, CA, USA) according to the manufacturer’s instructions. In brief, lymphocytes obtained from the spleens were incubated with PMA (100 ng/mL)/Inomycin (1 μg/mL) and monensin (1 μg/mL) in complete media at 37 °C for 4 h. Surface staining was performed with a CD4-FITC for 15 min at 4 °C. The cells were fixed and permeabilized with fixation buffer and permeabilization wash buffer, and intracellular cytokine staining was performed with IL-17A-APC, IL-4-APC, FOXP3-PE, and IFN-γ-PE, respectively, for 20 min. Then cells were then analyzed by FACS.

### 3.11. Statistical Analysis

Data are expressed as mean ± standard deviation (SD). All experiments were performed in triplicate. Data were statistically evaluated by one-way ANOVA followed by Dunnett’s test for multiple comparisons. Significance was set at *p* < 0.05.

## 4. Conclusions

CN30, a 30% ethanol extract of *C. nutans* exhibited antitumor activity in HepA tumor-bearing mouse models at doses of 3 and 10 mg/kg, with a higher inhibition rate than that observed in the fluorouracil-treated mice as positive control. Furthermore, CN30 could significantly increase the thymus indices and IL-2 and IFN-γ levels in the serum. The results suggest that *C. nutans* has potential antitumor and immunomodulatory properties and CN30 may possibly have an indirect antitumor activity by enhancing immunologic functions, which would be explored as a potential therapeutic treatment against cancer in the future.
